# Supplementary material for: Home Healthcare Among Aging Migrants: A Joanna Briggs Institute Scoping Review
Source: Healthcare (Basel). 2025 Apr 10;13(8):863. doi: 10.3390/healthcare13080863 (PMC12027206; doi:10.3390/healthcare13080863)
Supplement: Supplementary file 1 [file healthcare-13-00863-s001.zip › healthcare-3518242-S1.pdf]

Supplementary Material S1: Search Strategy

(migr* OR immigrant* OR refugee* OR "asylum seeker*" OR "displaced person*" OR "foreign born" OR "migrant worker*" OR "immigrant population") AND ("older adult*" OR elder* OR senior* OR geriatric* OR "older people" OR "aged 65+" OR "aging population" OR "elderly population" OR "older individuals") AND ("home healthcare" OR "home care" OR "home health service*" OR "community-based care" OR "in-home nursing care" OR "remote health management" OR "home nursing" OR "home support service*" OR "in-home health service*") AND ("accessibility" OR utilization OR "health outcome*" OR barrier* OR challenge* OR practice* OR facilitator* OR "health disparity" OR equity OR service* OR "care provision") NOT (pediatric* OR "children" OR "young adult*")
