# Supplementary material for: Home Healthcare Among Aging Migrants: A Joanna Briggs Institute Scoping Review
Source: Healthcare (Basel). 2025 Apr 10;13(8):863. doi: 10.3390/healthcare13080863 (PMC12027206; doi:10.3390/healthcare13080863)
Supplement: Supplementary file 1 [file healthcare-13-00863-s001.zip › healthcare-3518242-S2.pdf]

Supplementary Materials S2: Characteristics of Included Studies

Study	Country	Setting/context	Participant characteristics	Outcomes measured	Description of main results
Blaakilde AL. 2015.	Denmark	Danish retirement migrants regarding their experiences with the need for care or support while living abroad	Danish retired migrants living in Spain aged 54-88, living on a social or private pension.<br>Danish retired migrants living in Turkey aged 41-79.	Find cases that illuminate the experience of living abroad and reveal factors that impede "residential normalcy", with a focus on issues related to health, need of care and place of residency.	**Accessibility and Cultural Competency:**<br>-policy of Denmark and EU do not support transnational living for Danish Retirees. Migration is accompanied by a withdrawal of benefits that are djfj Provided to residential citizens of Denmark<br>**Social and Community Support Systems:**<br>-no security for caretaking among friends and acquaintances, which might Have been a solution of lack of public support and adaptation of the Souther European traditions of care.<br>-most migrants return to their homeland when they become ill and frail<br>**Health Outcomes and Well-being:**<br>-warm weather is beneficial for health compared to Denmark<br>-new relationships rarely make up for family support and care provision<br>-as they grow older in their new settings, they lose energy and become discouraged in helping others<br>**Utilizations, barriers and challenges:**

					-Supplementary private insurance plans offered higher quality service and English speaking staff
Lai DWL. 2004.	Canada	Level and predictors of using selected home care services by elderly Chinese immigrants in Canada	Chinese immigrants in Canada aged 65 years and older.	Aims to bridge the knowledge gap by studying predicting factors of home care service utilization by one aging ethnic minority group.	**Accessibility and Cultural Competency:**<br>-living alone results in higher chance for elderly Chinese to use home care<br>-English language was not significant in predicting home care use<br>**Social and Community Support Systems:**<br>-Family members usually perform tasks of providing personal care to elderly Chinese (culture)<br>**Health Outcomes and Well-being:**<br>-relationship between Chinese health beliefs and physical health indicated That higher level of Chinese health beliefs was associated with poorer Physical health<br>-relationship between Chinese health beliefs and use of home care may have Been confounded by the physical health status of participants<br>**Utilizations, barriers and challenges:**<br>- people with stronger social support were more likely to use home care<br>-people with stronger agreement with Chinese health beliefs reported a high Probability of using home care

					-people with higher level of education reported better self-rated English Competency and vice versa
Brotman S. 2003.	Canada	Eldercare, a publicly-funded organization which provides elder care services in Ontario, between 1998-1999.	10 older ethnic women aged between 67-83 years and 3 family members<br>(Greek. Italian, South Asian, Black, Chinese)<br>16 staff of the organization Eldercare<br>14 people who worked in the community	Addresses the experience of access among ethnic minority elderly women	**Accessibility and Cultural Competency:**<br>-daughters provided most of the hands-on service provision, and sons were Also often actively involved in instrumental activities such as interpretation And system linking.<br>-older ethnic minority women require their children's help due to institutiona Demands for cultural and language interpretation<br>**Social and Community Support Systems:**<br>-combined effects of the rise of the proportion of elderly people in the Population, restructuring hospitals resulting in shorter bed stays and cutback In community health and social services have occurred over past years<br>**Health Outcomes and Well-being:**<br>-older women's perception that they are burdening their children may have An effect on their health<br>**Utilizations, barriers and challenges:**<br>-loss of autonomy and their families may find that their issues and needs are

					Least likely heard within the system in the current climate (especially women Of colour)<br>- children may have less time available to engage in support of elderly family members and face greater barriers to economic security
Litwin H. 2004.	Israel	Utilization of formal care services of older people in Israel	3403 older people in Israel, aged 60 years or more (Arab, Jewish, Russian)	Relationships between support networks, ethnicity, and utilization of formal care services.	**Accessibility and Cultural Competency:**<br>-social networks may encourage utilization of formal assistance by advocating On behalf of their members and by actively seeking care services for them<br>**Social and Community Support Systems:**<br>-neighbour-focused, restricted, and diverse network types all made greater Use of formal home-care than community-clan networks<br>-people in neighbour-focused groups were most likely to use publicly-funded Formal home-care<br>**Health Outcomes and Well-being:**<br>-most functionally disabled group and the oldest age group were the most Likely to utilize public home-care<br>**Utilizations, barriers and challenges:**<br>-limited availability of formal care services might result in low utilization

					Among the very people who lack the financial resources necessary to Overcome the barriers to accessibility
Nielsen LS, Angus JE, Howell D, Husain A, Gastaldo D. 2015.	Canada	Palliative home care	4 Chinese immigrants with terminal cancer, their family caregivers, and home care nurses	Discursive tensions present in home care policies when providing palliative home care to Chinese immigrants	**Accessibility and Cultural Competency:**<br>- Healthcare providers (HCPs) often face tension between addressing shared cultural beliefs (cultural competence) and prioritizing individual needs (patient-centered care). This mismatch highlights the risk of stereotyping while attempting to respect cultural differences, emphasizing the need for nuanced, critical interpretations of "culture" in practice.<br>- The practice of matching HCPs and patients based on shared language or race often overlooks contextual nuances, such as dialects or expertise in palliative care. This approach can perpetuate cultural essentialism and reduce care quality, underscoring the importance of moving beyond simplistic cultural assumptions to focus on individualized, context-sensitive care.<br>**Social and Community Support Systems: N/A**

					**Health Outcomes and Well-being: N/A**<br>**Utilizations, barriers and challenges:**<br>-language barrier has a cascading effect, which requires effective negotiation<br>And management in many facets of life
Miner S, McDonald MV, Squires A. 2018.	United States	Home health care services for Somali older adults in the United States	Somali older adults and family members living in NY Rochester aged over 50 years.	Explore and describe Somali older adults' and their families' perceptions of and experiences with HHC services in order to improve its use and access	**Accessibility and Cultural Competency:**<br>-advantageous for older adults with limited mobility and decreased access to<br>Transportation<br>-Somalis thought that HHC staff needed better training around communicatio<br>And awareness about Somali cultural practice<br>-stated that they need more support from the health system to fulfill their<br>Traditional caregiving roles<br>**Social and Community Support Systems:**<br>-HHC professional was described as an advocate, however there were cases<br>Where there was failure to develop trusting relationships<br>-Somali community's role: advocate<br>**Health Outcomes and Well-being:**<br>-HHC is useful for learning about medications and managing chronic

					Illnesses<br>-HHC is identified as a resource for improvid<br>**Utilizations, barriers and challenges:**<br>-lack of understanding of their availability and purpose
Chen X, Frennert S, Östlund B 2022.	N/A	Home care and home care technologies for aging populations including older immigrants	N/A	To map existing knowledge of older adult immigrants' use of information and communication technologies for home care service published in scientific literature from 2014 to 2020.	**Accessibility and Cultural Competency:**<br>-involvement of a family members<br>-social and political support could improve the affordability and accessibility<br>Of ICT infrastructure and devices to older immigrants<br>-older immigrants expressed a greater willingness to use technology if the<br>Technology in question could be tailoured to cultural preferences that fit thei<br>Needs.<br>**Social and Community Support Systems:**<br>-social support and health status did not related to e-Health literacy<br>-education, social support and familiarity with the internet could improve the<br>Patient portal's using experience and frequency<br>-the social support should consider the older adult's use behaviour of the<br>Internet and assist the web designer in improving the web

					**Health Outcomes and Well-being:**<br>**Utilizations, barriers and challenges:**<br>-low SES<br>-insufficient language proficiency<br>-social integration due to limited scope of social networking
Knipping D, Garnett A, Jiang BB. 2023.	N/A	Home- and community-based services (HCBS) in westernized countries	N/A	Identify facilitators and barriers to access and use of HCBS for informal caregivers of culturally and linguistically diverse (CLD) older adults	**Accessibility and Cultural Competency:**<br>-Caregivers' knowledge about HCBS<br>-knowledge came from HCPs who shared their CLD identity or understood<br>The barriers they faced<br>-preferred culturally tailoured HCBS or programs with culturally diverse<br>Participants, but it was not always accessible due to long waitlists, limited<br>Ability of these services to meet care needs, or nonavailability<br>**Social and Community Support Systems:**<br>-in the absence of accessible formal sources of knowledge, desperation,<br>Informal networks, persistence and luck facilitated access information,<br>Such as friends or family with insider knowledge of HCBS<br>**Health Outcomes and Well-being:**

					**Utilizations, barriers and challenges:**<br>-difficulty navigating health care system and difficulty understanding Information about HCBS<br>-delayed dementia<br>-beliefs of caregivers, care recipients, and their community about caregiving and dementia, and previous negative individual or collective experiences with health and social services
Curtin, A., Martins, D. C., Gillsjö, C., & Schwartz-Barcott, D. 2017.	United States	Meaning of home care for older Hispanic immigrants in the United States	17 Hispanic participants, ranging in age from 65 to 83 years	Explore the meaning of home care among older Hispanic immigrants who are "aging out" of place"	**Accessibility and Cultural Competency:**<br>-there is a need to develop innovative care models which recognize the Importance of family relationships and a community which shares similar Social and cultural values in the provision of care in home setting<br>**Social and Community Support Systems:**<br>-meaning of home for Hispanic immigrants is 'close-knit', 'loving' families.<br>-participants had more a social life in their homeland compared to the U.S<br>-low language proficiency leads to less communication ? social isolation ? lack of accessibility<br>-lack of sense of community within their current neighborhoods.

					**Health Outcomes and Well-being: N/A**<br>**Utilizations, barriers and challenges (embedded in social and community Support systems)**
Zhao M, Zhang H, Lin X, You E, Wang H, Lautenschlager NT. 2023.	Australia	Non-immigrants and Chinese immigrants living in Melbourne and their perception and attitude towards dementia	21 Chinese immigrants living in Melbourne and 25 Chinese people living in Beijing who did not have a dementia diagnosis	Understand what older Chinese adults think about dementia and to inform the development of tailoured dementia-related services for this group.	**Accessibility and Cultural Competency:**<br>-stigma and stereotypes still present for some people about dementia<br>-home care is preferred, but formal care is the mainstream form of care in Future<br>-being interested in dementia educational activities increase accessibility<br>**Social and Community Support Systems:**<br>-tragedy for the whole family, especially for adult children<br>-family members and community members being willing to help people living with dementia<br>-avoid the topic of dementia and people living with dementia<br>**Health Outcomes and Well-being:**<br>-a scary and hopeless condition<br>-unpleasant topic<br>-being worried about developing dementia

					**Utilizations, barriers and challenges:**<br>-family responsibilities, taking care of their grandchildren, helping with housework<br>-Transportation problems, inability to drive, inconvenient public transportation<br>-low English proficiency
Yang K, Chao Y-Y, Zha P, Wang W, Lee Y-S. 2022.	United States	Cultural dynamics and relationships between home health aides and Chinese immigrants	Older Chinese immigrants and their home health aids	To explore caregiving and receiving experiences among older Chinese immigrants and their home health aids in culturally concordant dyads in a community setting	**Accessibility and Cultural Competency:**<br>-participants expressed difficulties in finding a caregiver of the same culture<br>-most care recipients mentioned they had ethnic food preferences<br>**Social and Community Support Systems:**<br>-relationships between caregivers and clients were described as companionship and rapport<br>-promotes older adult's social interactions<br>**Health Outcomes and Well-being:**<br>-caregivers reminded their clients to take medications and accompanied them to doctor's appointment<br>-provided advice for disease management, assisted in daily routines to prevent falls and promote healthy behaviors

					-combining home care with professional care resulting in better health outcomes<br>**Utilizations, barriers and challenges:**<br>-caregiver’s burden while serving dual roles as paid caregiver and family<br>Member<br>-not being able to find a caregiver who does not have the same culture
Carlsson H. 2023.	The Netherland s	Home care, home aid, day care, local minority communities	Older migrants who were residents of Nijmegen, The Hague	Identify relationships of care that facilitate access to aged care for older first-generation migrants	**Accessibility and Cultural Competency:**<br>-place of residence<br>-availability of services in their language (minority specific services)<br>-minority community facilitated access to care<br>**Social and Community Support Systems:**<br>-minority specific services are an important resource in negotiating the need<br>for social support in old age<br>-belonging to a local minority community is an important source of informal<br>Care and support for older migrants<br>**Health Outcomes and Well-being:**<br>-informal relationships of care enable health<br>**Utilizations, barriers and challenges:**

					-low language proficiency, illiteracy
van den Brink Y. 2003.	The Netherlands	Transcultural Care for the Elderly at Home	Turkish caregivers and Dutch professional nurses in the community of The Netherlands	Explore diversity and universality in care values and meaning relevant to care of the elderly at home.	**Accessibility and Cultural Competency:**<br>-When adult family members are not informed about the purpose of the Initial visit and the professional expectations, access to professional services At home may be refused by elders despite their need<br>-The need for culturally and linguistically congruent interpreters is Paramount to decrease the burden on family caregivers<br>**Social and Community Support Systems:**<br>-In Turkish culture, presence of available adult children is a very important Factor in meeting the needs of the parents<br>-Nurses relieve family members' burden by informing them about social Services such as rules for eligibility, access, application<br>-Care from family includes emotional, financial, and social support through Regular visits, phone calls, sending money or medicines<br>-adult children accompany their parents to appointments, and act as translators between the parents and the HCPs<br>**Health Outcomes and Well-being:**

					-professional caregivers should negotiate with the family in accommodating Their generic treatments, thus preventing them from hiding their activities from professional caregivers, which can have an impact on their health<br>**Utilizations, barriers and challenges:**<br>-absence of adult children who are available
Denktas, S., Koopmans, G., Birnie, E., Foets, M., & Bonsel, G. 2009.	The Netherland s	Health care access for older migrants in The Netherlands	First-generation immigrants aged 55 years and older in The Netherlands	Assess whether ethnic disparities in health care use exist and how they can be explained	**Accessibility and Cultural Competency:**<br>-use of GP services is higher among all immigrant groups, while use of PT And home care is low to absent<br>-health status and language competence have high explanatory power for all Types of health services utilization<br>-there is a resulting pattern of systematic lower health services utilization of Elderly immigrants<br>**Social and Community Support Systems:**<br>-Turkish and Moroccan group more often have a traditional attitude on family Care, male/female roles and family values<br>**Health Outcomes and Well-being:**<br>-poor self-assessed health and more chronic conditions<br>-self-report of poor mental health

					**Utilizations, barriers and challenges:**<br>-health disparities are aggravated by lack of language competence
Bolzman C, Vagni G. 2017.	Switzerland	Access to home care from private services or informal care for older migrants from Italy, Spain, Portugal	Older migrants from Italy, Spain, Portugal living in Switzerland, aged 65 to 69.	Explore help and home-care services together with other ambulatory care activities provided mainly by the public sector and not-for-profit organizations or by private services, and the informal care provided by family and friends.	**Accessibility and Cultural Competency:**<br>-in spite of many disadvantages as a migrant, older immigrants find their Way to these services as much as do Swiss natives and citizens.<br>-whether you hold a Swiss citizenship or not makes a difference in access to Care services<br>**Social and Community Support Systems:**<br>-those who receive informal care help from friends and neighbours are also Using more social care services<br>-they do not look exclusively for support within their family and informal Networks.<br>**Health Outcomes and Well-being:**<br>-poor health related to difficult working conditions before retirement and Fraility related to their age are decisive factors in explaining the use of public and non-profit care services<br>-Portuguese are seriously concerned with poor health and precarious life Conditions, therefore, seek social care services

					-those who are in poor health make more use of these services<br>**Utilizations, barriers and challenges:**<br>-elderly who hold a university degree use these services more frequently<br>Regardless of their marital status.<br>- those who are divorced, separated, or widowed use more social care services than those who are married
Kuo T, Torres-Gil FM. 2001.	United States	Access to health care services and home- and community-based care programs	Older Taiwanese elderly people living in Southern California	Examine factors that affect the utilization of health care services and home- and community-based care (HCBC) programs by older Taiwanese, a rapidly growing and newly arrived immigrant group.	**Accessibility and Cultural Competency:**<br>-use of these programs was influenced by years of immigration, living alone,<br>Preferring services by professionals of the same cultural background<br>**Social and Community Support Systems:**<br>-A lot of Taiwanese elderly people do not have health insurance.<br>-emergency funds are set up by organizations such as Tzu-Chi or the<br>Taiwanese American Affiliated Committee and Aging, but limited<br>**Health Outcomes and Well-being:**<br>-Taiwanese association that are targeted for older Taiwanese immigrants<br>Provided social support for older immigrants who live alone or in isolation<br>To meet their peers and engage in social activities that that are culturally<br>And linguistically appropriate.

					**Utilizations, barriers and challenges:**<br>-affordability, availability, accessibility of services, health literacy, social Support networks, position in society all impact the access to health care Services and home care.
Graham CL, Ivey SL, Neuhauser L. 2009.	United States	Transition from hospital to home	Older adults in one ethnically and socioeconomically diverse region: San Francisco Bay Area	Assess the needs of patients and caregivers during the transition from hospital to home	**Accessibility and Cultural Competency:**<br>-caregivers and seniors relied on informal support rather than paid service Agencies<br>-filial piety: certain ethnic groups reported having fewer options for using Formal LTC services<br>-there is lack of linguistically appropriate information and services<br>**Social and Community Support Systems:**<br>-presence of family members<br>-reported feeling of isolation and unsupported during a health care transition Because their informal support networks had been compromised by immigrant<br>**Health Outcomes and Well-being:**<br>--recent immigrants and LGBT seniors can be at risk or preventable Rehospitalization due to lack of informal support, which often is exacerbated By the fact that HCPs fail to recognize the problem

					-seniors with lower SES have higher rates of hospitalization and mortality After hospital discharge<br>**Utilizations, barriers and challenges:**<br>-inadequate caregiver training of ADLs<br>-inadequate information from discharge planners<br>-inability to afford personal care services, to find affordable and accessible Housing, to afford home modifications
Fernandes R, Braun KL, Ozawa J, Compton M, Guzman C, Somogyi-Zalud E. 2010.	United States	Kokua Kalihi Valley Comprehensive Family Services, which is a Home-Based Palliative Care Program in Honolulu.	Ethnic minority populations, with chronic advanced illnesses	Measure symptom relief and quality of life, resource utilization, and satisfaction with HBPC.	**Accessibility and Cultural Competency:**<br>-the program revealed that there was an increase in appropriate use of Community resources of this population<br>-team comprising of bilingual case managers was key to building trust in the health care system by bridging cultural and language gaps<br>**Social and Community Support Systems:**<br>-mean satisfaction rankings were 4.5 for emotional and spiritual support for Assistance with financial issues<br>-the program links the client and support groups if the client wishes to have Companionship.

					**Health Outcomes and Well-being:**<br>-stabilize the care of seriously and terminally ill patients at home<br>-minimize pain and anxiety for most clients<br>-reduce hospitalizations<br>**Utilizations, barriers and challenges:**<br>-this population present challenges that arise from the complex interplay of Advanced illness, low functional status, limited English skills, low health Literacy, poverty, and cultural consideration
Lendon JP, Rome V, Sengupta M. 2021.	United States.	Adult Day Service Centers (ADSCs) in various U.S. regions, including urban and rural areas.	-The data are collected from ADSC directors or managers at the provider level and contain information about ADSCs and aggregate-level characteristics about participants.<br>- Aggregate participant	- The study measured the differences in characteristics of ADSCs and participants based on racial and ethnic case-mix.<br>-It looked at service provisions, health conditions,	**Accessibility and Cultural Competency:**<br>- Minority accommodating ADSCs were more likely to provide transportation services.<br>- Programming for diabetes was more common in predominantly minority centers.<br><br>**Social and Community Support Systems:**<br>- For-profit centers were more likely to serve predominantly racial and ethnic minority groups.

			characteristics included the percentage of participants per center with these characteristics: female; aged 65 and older; difficulty eating, dressing, bathing, toileting, and walking; and with each of the following diagnosed health conditions: intellectual or developmental disabilities (IDD), Alzheimer's disease or other dementias, severe mental illness, depression, cardiovascular	operational characteristics, and demographic attributes.	**Health Outcomes and Well-being:**<br>- Higher prevalence of diabetes among participants in minority-serving centers.<br>- Lower percentages of participants with Alzheimer's or dementia compared to predominantly White centers.<br><br>**Utilizations, barriers and challenges:**<br>- Minority-serving ADSCs had lower self-pay revenue and were more dependent on Medicaid.<br>- Barriers such as fewer programs for cardiovascular disease in non-Hispanic Black-serving centers were noted.

			disease, and diabetes.		
Conkova N, van den Broek T. 2024.	The Netherlands.	Focus on older migrants across the Netherlands (assumed both rural and urban areas) , examining their use of the long-term care (LTC) system.	-Foreign-born individuals aged 55+ in the Netherlands.<br>-These include migrants of Western and non-Western origins, with specific groups, such as those of Turkish, Moroccan, Surinamese, Antillean, and other non-Western origins.	-The study explored the current and projected use of LTC provisions (residential care, home-based care, and personal care budgets) by older migrants in the Netherlands, as well as barriers and facilitators for LTC access.	**Accessibility and Cultural Competency:**<br>- Turkish and Moroccan migrants showed lower usage of residential care, possibly due to cultural preferences for family-based caregiving.<br>- Increased use of home-based care and personal care budgets was observed among these groups.<br><br>**Social and Community Support Systems:**<br>- Reliance on informal care and family support remains significant among Turkish and Moroccan older adults.<br>- Western migrants exhibited higher uptake of formal LTC services, indicating varying levels of integration into the LTC system.<br>-<br>**Health Outcomes and Well-being:**<br>- Not directly addressed in terms of specific health outcomes but

					inferred through LTC utilization trends. Home-based care was seen as potentially better tailored to meet cultural and religious needs.<br>**Utilizations, barriers and challenges:**<br>- Barriers included language difficulties, lack of awareness about LTC systems, and cultural stigma around formal care.<br>- The demand for culturally sensitive care was highlighted as a pressing need for effective home-based LTC delivery.
White L, Klinner C. 2012.	Australia.	Suburban areas of Sydney, New South Wales, with a focus on community settings for elderly Chinese and Vietnamese immigrants.	-Elderly Chinese and Vietnamese immigrants aged 55 -83 years, with poor English proficiency, eligible for the Home Medicines Review (HMR).<br>- Participants were defined as belonging to the respective culturally and linguistically	The study explored medication management practices, awareness of the HMR program, and participants' attitudes towards its use.	**Accessibility and Cultural Competency:**<br>- Limited English proficiency and a lack of bilingual pharmacists were significant barriers to accessing medicine information.<br>- Participants expressed a need for culturally sensitive care, such as having HMR pharmacists who spoke their language or provided interpreters.<br>**Social and Community Support Systems:**<br>- Many participants relied on family, friends, or community associations for advice due to dissatisfaction with formal healthcare providers.<br>- Chinese participants reported skepticism towards their general

			diverse (CALD) group if they self-identified as belonging to the respective group, spoke their native language at home and had limited English skills.		practitioners (GPs), often modifying medication dosages without informing their doctors.<br><br>**Health Outcomes and Well-being:**<br>- N/A (The study primarily focused on attitudes and access instead of specific health outcomes.)<br>**Utilizations, barriers and challenges:**<br>- Awareness of the HMR program was low, with none of the participants having prior knowledge of it.<br>- Concerns about involving GPs in the HMR process were significant especially among Chinese participants, who feared it might upset their doctors.<br>- Vietnamese participants displayed greater trust in their GPs and were open to discussing the program with them.
Miner SM, Liebel D, Wilde MH, Carroll JK, Zicari E,	United States.	Primarily urban settings (Rochester, New York)	-Older adult refugees from diverse backgrounds, predominantly	-Pain management<br>-Anxiety level<br>-Depression	**Accessibility and Cultural Competency:**<br>- HHC services successfully implemented culturally sensitive care models incorporating emic (client's cultural perspective) and etic (provider's

Chalupa S. 2017.			from Somalia and Nepal, with smaller representation from countries like Burma, Liberia, Vietnam, Cambodia, and Burundi.<br>-Most participants were female (65%), aged 32–96, and covered by Medicaid.	-Medication management<br>-Activities of daily living (ADLs)<br>-Health service utilization	perspective) care views.<br>- Refugees improved their ability to manage Western medications and benefited from culturally competent education strategies, addressing linguistic and cultural barriers.<br>**Social and Community Support Systems:**<br>- Participants predominantly lived with family (88%), suggesting strong familial support systems that may influence the uptake and impact of HHC services.<br>**Health Outcomes and Well-being:**<br>- Significant improvements in anxiety levels, pain interference, ADL management, and medication management were reported.<br>For example:<br>- Anxiety scores decreased from a median of 1.00 to 0.00.<br>- Pain scores dropped from 3.00 to 1.00.<br>- Improved functional independence in ADLs and medication management.<br>- Depression screening revealed potential underdiagnosis due to

					cultural or tool-related limitations.<br>**Utilizations, barriers and challenges:**<br>- Underutilization of telehealth and home health aide services, possibly due to cultural norms prioritizing family caregiving.<br>- Frequent use of skilled nursing, physical therapy, and occupational therapy (higher than national averages).<br>- Limited understanding of Western healthcare practices and transportation barriers prior to the intervention.
Kim BJ. 2011.	United States.	Adult Day Health Care Centres (specifically Los Angeles, California), urban setting.	Korean immigrants, aged 65 and older, who resided in Los Angeles County. Participants were interviewed once at adult day health care centers where they received services.	Explored the impacts of individual characteristics (socioeconomic status, cognitive functioning, and physical health), social environment (family support), physical environments (number of days and years attending	**Accessibility and Cultural Competency:**<br>- Many low- income elderly Korean immigrants faced challenges in culturally competent social services despite receiving basic medical services through Medicaid or Medicare.<br>- ADHC centers provided culturally tailored medical and social services, improving access to necessary care<br>**Social and Community Support Systems:**<br>- Strong family support was directly and indirectly associated with a higher QOL.<br>- Participants with stronger family support were more likely to attend

				ADHC), and quality of life (physical QOL, mental QOL, and reported health transition) on ADHC use for elderly Korean Immigrants.	ADHC for longer durations, which enhanced their QOL<br>**Health Outcomes and Well-being:**<br>- Longer attendance at ADHC positively correlated with better physical and mental health components of QOL.<br>- Family support and longer participation in ADHC contributed to improved cognitive and mental health among participants.<br>**Utilizations, barriers and challenges:**<br>- Language barriers and lack of cultural competency in broader healthcare services remained challenges.<br>- The demand of culturally-adjusted and disability accommodated ADHC in Metropolitan Los Angeles areas is dramatically high due to high population rate of minority elderly.<br>- ADHC centers addressed these challenges but relied on public funding to do which caused budget threats

Miner S. 2016.	United States.	Urban setting, specifically in Rochester, New York, where a large Somali refugee population resides.	-The study included 14 Somali families (19 individuals), comprising older adults (aged 50 and above) and their caregivers.<br><br>-Participants had varied lengths of residency in the U.S., ranging from less than one year to 34 years.	The study explored Somalian family perceptions and experiences with adult home health care (HHC) services to understand how to improve access, usage, and effectiveness of these services.	**Accessibility and Cultural Competency:**<br>- Somali families found HHC beneficial but identified cultural and linguistic barriers.<br>- Families wanted HHC professionals to understand Somali culture and refugee experiences, including practices like prayer times and removing shoes indoors.<br>- Lack of awareness about HHC services was a significant barrier to access.<br>**Social and Community Support Systems:**<br>- Somali families emphasized the importance of community involvement in facilitating access to HHC.<br>- They wanted community leaders to collaborate with HHC agencies to enhance understanding and communication.<br><br>**Health Outcomes and Well-being:**<br>- HHC improved health literacy by teaching older adults and caregivers about medication management and chronic illness care.<br>- Families reported that having HHC at home reduced stress and

					improved the health outcomes of older adults by providing personalized care in a familiar setting.<br><br>**Utilizations, barriers and challenges:**<br>- Families preferred caregiving at home but faced challenges like economic limitations and unclear program requirements.<br>- Consumer-Directed Personal Assistance Programs (CDPs) were valued for allowing families to provide culturally congruent care, but issues like limited-service hours and confusion about eligibility caused dissatisfaction.<br>- Trust in HHC professionals significantly influenced families' satisfaction, with trust-enhancing experiences fostering better health outcomes.
Hansen E. 2014.	Denmark	Urban settings (Municipality of Copenhagen).	-Older adults aged 65+ residing in Copenhagen, including ethnic Danes and immigrants from both Western and non-Western countries.	-Use of municipal long-term care services (home care, personal care, assistance with domestic chores, residential care).	**Accessibility and Cultural Competency:**<br>-Non-Western immigrants had lower usage of municipal care services compared to ethnic Danes and Western immigrants, likely due to language barriers and limited knowledge of the Danish welfare system.<br><br>-Increased length of residence improved immigrants' knowledge and use of

			-Participants were categorized by length of residence in Denmark (<10 years or >10 years).<br><br>- Non-Western immigrants included those from countries such as Turkey, Pakistan, Iraq, and Iran.	-Factors influencing care utilization (socioeconomic status, cultural practices, and length of residence).	services, indicating growing cultural assimilation over time.<br>**Social and Community Support Systems:**<br>-Informal care played a significant role among non-Western immigrants, with family members often assuming caregiving responsibilities, especially for domestic chores.<br>-Cultural norms favoring family-based care influenced lower utilization of municipal services.<br><br>**Health Outcomes and Well-being:**<br>- N/A (health outcomes such as quality of life or specific health improvements were not directly assessed).<br>**Utilizations, barriers and challenges:**<br>- Language difficulties, lack of awareness of available services, and reliance on informal care.<br>- Non-Western immigrants utilized services like domestic chores assistance and residential care far less frequently than ethnic Danes.<br>- For example: Only 14% of non-Western immigrants (<10 years in Denmark) used any municipal long-term care services, compared to 40% of

					ethnic Danes.<br>- Adoption of Western caregiving norms were slower among non-Western immigrants, though this gap narrowed with longer residence.
Xiao LD, Willis E, Harrington A, Gillham D, De Bellis A, Morey W, et al. 2018.	Australia.	Aged care homes, urban setting.	- Elderly individuals in aged care homes, including culturally and linguistically diverse (CALD) residents and non-CALD residents.<br>-Care workers from CALD and non-CALD backgrounds, including managers and nurses.<br>-Family members of the residents who acted as	- Cross-cultural communication (CCC) challenges and strategies in aged care homes.<br>-Factors that influence effective CCC between staff, residents, and families.<br>-Development of culturally appropriate communication strategies.	**Accessibility and Cultural Competency:**<br>- Staff demonstrated cultural humility, which improved communication and empowered clients from CALD background.<br>- CALD staff faced challenges due to language and cultural barriers.<br>**Social and Community Support Systems:**<br>- Families played a role in bridging communication gaps in bridging communication gaps by acting as interpreters or providing communication aids.<br>- Collaboration between residents, families and staff helped develop personalized communication resources.<br>**Health Outcomes and Well-being:**<br>- Empowering CALD residents in communication contributed to their psychological well-being.<br>- Staff efforts to acknowledge and learn of residents' cultural and

			communication facilitators.		linguistic needs positively impacted care experience.<br>**Utilizations, barriers and challenges:**<br>- Language proficiency among CALD staff and residents posed barriers to effective communication.
Songur W. 2022.	Sweden.	Urban settings, focusing on Sweden's three largest cities: Stockholm, Malmö, and Gothenburg.	Older migrants aged 65+, including individuals from the Middle East and Africa, as well as native Swedes and other European migrants.	- Utilization of home help services and nursing homes.<br><br>- The role of privatization and ethnically-profiled care in improving accessibility for older migrants.<br><br>-Focuses on how different groups utilize elderly care services, particularly home help services and nursing homes.	**Accessibility and Cultural Competency:**<br>- Ethnically profiled elderly care services (e.g., care in native languages) were more accessible in Stockholm, where privatization was extensive.<br>- Older migrants, especially from the Middle East and Africa, had greater access to culturally and linguistically adapted services in Stockholm compared to Malmö and Gothenburg.<br>- The "system of choice" in Stockholm facilitated personalized care, attracting private providers to meet specific cultural needs.<br><br>**Social and Community Support Systems:**<br>- Highlights the importance of cultural and linguistic connections in fostering a sense of belonging among older migrants.<br>- Migrants from the Middle East and Africa benefited from informal

					community networks in conjunction with formal care services.<br><br>**Health Outcomes and Well-being:**<br>- N/A (The study does not directly investigate health outcomes or well-being).<br>**Utilizations, barriers and challenges:**<br>- Older migrants from the Middle East and Africa used home help services more in Stockholm compared to Malmö and Gothenburg. Nursing home use among these groups remained lower than among native Swedes, but access was better in Stockholm due to the availability of ethnically-profiled nursing homes.<br><br>- Language, cultural preferences, and reliance on informal family support limited the use of nursing homes, particularly in Malmö and Gothenburg.<br>- Variability in privatization strategies across cities led to unequal access to care, with Stockholm outperforming Malmö and Gothenburg in accommodating older migrants' needs.

Songur W. 2019.	Sweden.	Urban settings, focusing on large cities with high migrant populations.	Older migrants aged 65+ residing in Sweden, specifically migrants from the Middle East and Africa, along with native Swedes and migrants from Nordic and European countries.	-Examined preferences and use of home help services and special housing.<br>- Usage of elderly care services, including home help services and special housing.<br>-The influence of family support and cultural norms on elderly care choices.	**-Accessibility and Cultural Competency:**<br>- Older migrants from the Middle East and Africa used home help services at rates similar to native Swedes, but they avoided special housing.<br><br>-Families played a critical role in enabling access to and understanding of home help services, compensating for language barriers and system unfamiliarity.<br><br>**Social and Community Support Systems:**<br>- Strong family networks provided significant informal care for elderly migrants.<br>- Cultural expectations of filial piety influenced decisions, with families opting for home help services to complement their own caregiving, avoiding special housing which was culturally stigmatized.<br>**Health Outcomes and Well-being:**<br>- N/A (specific health outcomes were not directly measured).<br>**Utilizations, barriers and challenges:**<br>- Home help services were preferred by Middle Eastern and African

					migrants, while special housing was underutilized due to cultural preferences and stigma.<br>- Language difficulties and reluctance to access special housing services were major obstacles.<br>- Conflicts occasionally occur between family caregivers and care providers regarding the extent of care needs and service utilization.
de Graaff FM, Francke AL. 2003.	The Netherlands.	focused on the experiences of families in urban and mixed community settings, with some participants living in predominantly Turkish or Moroccan neighborhoods and others in predominantly Dutch areas.	The study included 19 respondents (family members of terminally ill Turkish and Moroccan patients) who had cared for their terminally ill relatives. Participants were split into users and non-users of professional home care services.	-The study explored the experiences of Turkish and Moroccan families with Dutch professional home care services during the terminal phase of illness.<br>- Aimed to identify factors affecting access to and use of these services.	**Accessibility and Cultural Competency:**<br>- Limited awareness about professional home care and its benefits was a barrier, with respondents often uninformed about available options.<br>- Language and cultural barriers made accessing care challenging; there was a demand for more care providers from Turkish and Moroccan backgrounds.<br>- Cultural norms, including preferences for family care and reluctance to allow "strangers" into the home, influenced care decisions.<br><br>**Social and Community Support Systems:**<br>- Social pressure within Turkish and Moroccan communities often dictated care decisions, prioritizing family caregiving over professional

					services.<br>- Community support varied; families in predominantly Dutch neighborhoods were more open to using professional services compared to those in immigrant neighborhoods.<br><br>**Health Outcomes and Well-being:**<br>- Families who used home care reported that professional support, such as nursing guidance and technical aids, enhanced the quality of care.<br>- Positive relationships with nurses improved satisfaction, though communication issues with general practitioners were highlighted.<br><br>**Utilizations, barriers and challenges:**<br>- Barriers included a lack of knowledge about home care, insufficient referrals by general practitioners, and cultural stigma around using professional services.<br>- Female family members often bore the brunt of caregiving, leading to stress and difficulty managing their other responsibilities.<br>- Decisions to use home care were influenced by family structure,

					community expectations, and the patient's or family's preference for care in their home country.
de Graaff FM, Francke AL. 2009.	The Neitherlands.	Urban settings, particularly areas with high populations of Turkish and Moroccan migrants.	-General practitioners (GPs) and home care nurses working in areas with high numbers of Turkish and Moroccan terminally ill patients.<br>-Terminally ill Turkish and Moroccan migrants and their families were the focus of the study, but data were collected from healthcare providers.	-Barriers to accessing and utilizing home care for terminally ill Turkish and Moroccan migrants.<br>-Perceptions of GPs and nurses regarding the provision of culturally competent care and communication challenges.<br>-Factors influencing the integration of professional home care	**Accessibility and Cultural Competency:**<br>- Communication issues were a significant barrier, stemming from language differences and cultural misunderstandings.<br>- Limited knowledge about home care services among migrant families affected their utilization.<br>- A preference for family caregiving over professional care was culturally rooted and influenced access to home care.<br><br>**Social and Community Support Systems:**<br>- Families of terminally ill patients often assumed caregiving roles, driven by cultural expectations of familial responsibility.<br>- Informal caregivers faced overburdening, leading to calls for better support systems.<br><br>**Health Outcomes and Well-being:**

				services in these communities.	- Professional home care improved the quality of end-of-life care when utilized, but it was inconsistently accessed.<br>- Patients who received home care showed better management of terminal conditions compared to those relying solely on family care.<br>**Utilizations, barriers and challenges:**<br>- GPs and nurses noted significant challenges, including difficulty in assessing patients' needs due to reliance on family translators.<br>- Cultural taboos around discussing terminal illnesses prevented open communication about care needs.<br>- Financial concerns and misunderstandings about payment for home care services further limited utilization.
Hierofani PY, van Riemsdijk M. 2024.	Sweden.	The study focuses on urban settings, specifically in the greater Stockholm and Uppsala areas, which have	-35 individuals comprising care recipients, family caregivers, municipal staff, and migrant organization representatives.	- Investigated types of family caregiving, challenges, institutional support needs, and personal experiences of care provision	**Accessibility and Cultural Competency:**<br>- Public care services in Sweden often lack cultural and linguistic sensitivity, making them less accessible to immigrant elderly.<br>- Immigrant-specific needs, like native language services and culturally familiar food, are not always met.<br>**Social and Community Support Systems:**

		large immigrant populations.	-Care recipients and caregivers were mostly female, aged 30s-80s, originating from various countries including Iran, Finland, Iraq, Syria, Turkey, Angola, and Spain.	among older immigrants.	- Family caregiving is often culturally expected, with a focus on reciprocity and filial duty.<br>- Migrant organizations play a role in connecting caregivers and recipients with support resources.<br>**Health Outcomes and Well-being:**<br>- Family caregiving contributes to the psychological well-being of older immigrants due to cultural familiarity.<br>- Lack of culturally sensitive public care can negatively affect the satisfaction and overall well-being of care recipients.<br>**Utilizations, barriers and challenges:**<br>- Barriers to public care include language issues, lack of cultural competency, and limited trust in public care providers.<br>- Family caregivers face challenges like balancing care duties with personal lives, insufficient support, and financial strain.<br>- Publicly funded support, such as financial compensation or respite care is often inadequate or inaccessible to immigrant families.
Lan P-C. 2002.	United States.	Urban setting, primarily within	Middle-class Taiwanese and	-Transformation of cultural	**Accessibility and Cultural Competency:**

		immigrant families’ homes.	Hong Kong immigrant families in California.<br><br>- Included aging parents (68–88 years old), adult children (38–60 years old), and home care workers (51 - 64 years old, mostly women from mainland China and Taiwan).	practices of filial piety among immigrant families in the U.S. context through 3 dimensions; where care takes places, who gives care, and who pays for care.<br><br>-The impact of caregiving arrangements on intergenerational relationships, traditions, and family dynamics.<br><br>.	- Elderly immigrant parents accessed caregiving services through public programs like In-Home Supportive Services (IHSS) and private hiring.<br><br>- Cultural factors, including the preference for caregivers of similar ethnic and linguistic backgrounds, influenced caregiving arrangements.<br><br>- Cultural Chinese traditions of having children of the family fulfill filial responsibilities for their aging parents can influence perspectives on elder care.<br>**Social and Community Support Systems:**<br>- Family remained central to caregiving networks, with adult children coordinate financial and caregiving responsibilities.<br>- The inclusion of caregivers as "fictive kin" helped preserve cultural ideals of filial piety despite outsourcing caregiving tasks.<br><br>**Health Outcomes and Well-being:**<br>- Caregiving arrangements improved elderly parents’ autonomy

					and well-being by providing personalized, culturally relevant care.<br>- Emotional and psychological benefits arose from maintaining family-like relationships with caregivers.<br>-<br>**Utilizations, barriers and challenges:**<br>- Challenges included the stigma of using public welfare programs, reliance on informal caregiving networks, and conflicts arising from cultural and class differences between families and caregivers.<br>- Financial burdens and time constraints for dual-earner families made private caregiving essential yet costly.
Andersson K, Johansson S. 2021.	Sweden.	Urban settings, focusing on elderly home care services.	-Elderly individuals with migrant backgrounds seeking home care services.<br>-Care managers (all women, with varying levels of education and work experience	-How care managers assess and address the needs of elderly migrants in home care services.<br>-Tensions between universal standards, local	**Accessibility and Cultural Competency:**<br>- Care managers found it challenging to balance standardized care models with the diverse cultural expectations of elderly migrants.<br>- Language barriers and limited understanding of the Swedish welfare system among migrants impacted access to services.<br>**Social and Community Support Systems:**

			in elderly care services).	guidelines, and individual needs.<br>-Challenges related to cultural diversity in the provision of care.	- Migrant families often sought next-of-kin employment to provide care for their elderly, reflecting cultural norms of family responsibility. Care managers perceived this as potential misuse of the welfare system, lead<br>**Health Outcomes and Well-being:**<br>N/A (No direct measurement of health outcomes was reported in the study).<br>**Utilizations, barriers and challenges:**<br>- Barriers included care managers' reliance on stereotypical views of migrants, lack of cultural competency, and inflexible adherence to local guidelines.<br>- Migrants' needs were often misunderstood or deprioritized due to systemic biases favoring Swedish-born clients.<br>- The concept of independence in Swedish welfare norms clashed with migrants' cultural values emphasizing interdependence within families.
Chaouni SB, Smetcoren AS,	Belgium.	Prominently urban, big focus	- 12 informal- a child or child-in-law or partner of a	-The study explored the care experiences of older	**Accessibility and Cultural Competency:**<br>- Language barriers and cultural differences posed significant

De Donder L. 2020.		on Belgian city settings	senior of Moroccan origin with dementia;<br>- Professional caregivers in the field of dementia care in several Belgian cities who have worked with Moroccan seniors.	Moroccan migrants with dementia in Belgium.<br>-It investigated how transnational networks of informal and professional care operate and the challenges faced.<br>-Specific focus areas included access to care, cultural perceptions of dementia, family dynamics, and interactions between informal and professional caregivers.	challenges in accessing professional care.<br>- There was a lack of culturally appropriate dementia care services, including language support and recognition of cultural norms around caregiving.<br>- Professional caregivers often lacked understanding of the Moroccan cultural context, creating mistrust and resistance from families.<br><br>**Social and Community Support Systems:**<br>- Caregiving networks often extended transnationally, with family members across Belgium and Morocco participating in decision-making and care provision.<br>- Moroccan families relied heavily on informal caregiving, with women (daughters or daughters-in-law) frequently taking primary responsibility.<br>- Community and religious organizations played limited roles in providing support, highlighting a gap in formal community systems.<br>**Health Outcomes and Well-being:**<br>- Families expressed significant emotional and physical stress, particularly among primary caregivers, due to the burden of care.

					- Older migrants with dementia often experienced isolation due to language and cultural barriers, affecting their overall well-being.<br>- Professional interventions, where utilized, improved patient outcome but were inconsistently accessed due to mistrust or lack of awareness.<br>**Utilizations, barriers and challenges:**<br>- Families preferred informal care due to cultural norms valuing family caregiving over institutional solutions.<br>- Limited awareness about available dementia care services led to underutilization of professional care.<br>- Financial constraints, legal issues (e.g., residency status), and inadequate information dissemination were significant barriers.<br>- Professional caregivers found it challenging to integrate into the caregiving process due to resistance from families and a lack of cultural training.
Cheung S-L, Barf H, Cummings S, Hobbelen H,	The Netherland s.	Urban settings, including cities like Groningen, Amsterdam,	-Second generation Chinese immigrants (aged	- Practices and perceptions of filial care among second	**Accessibility and Cultural Competency:**<br>- Participants acted as language brokers and intermediaries for their

Chui EW-T. 2020.		and other Dutch urban areas with a high Chinese immigrant population.	18–25) born or raised in the Netherlands, whose parents migrated from China, Hong Kong, Taiwan, or Macau.<br>-Participants were primarily students, divided equally between those living with their parents and those living independently.	generation Chinese immigrants.<br>-Influence of cultural heritage and filial piety on caregiving attitudes.<br>-Decision making regarding current and future care for aging parents.	parents in navigating formal care systems.<br>- Limited use of formal healthcare services by first-generation Chinese immigrants was noted, attributed to cultural preferences and language barriers.<br>- The younger generation facilitated access to services, reflecting modern adaptations of filial piety.<br><br>**Social and Community Support Systems:**<br>- Caregiving responsibilities were strongly influenced by familial expectations and the value of filial piety.<br>- Tasks such as home visits, facilitating appointments, and providing emotional support was common.<br>- Strong emphasis on family-based care rather than reliance on formal systems.<br><br>**Health Outcomes and Well-being:**<br>- N/A (Health outcomes were not directly assessed).<br>**Utilizations, barriers and challenges:**

					- Informal caregiving practices dominated, with participants bridging cultural and linguistic gaps for their parents.<br>- Cultural stigma around institutional care and insufficient language proficiency limited formal care use by first-generation immigrants.<br>- Participants balanced normative expectations of filial care with practical constraints, such as time and financial resources.
